# Health Literacy–Informed Communication to Reduce Discharge Medication Errors in Hospitalized Children: A Randomized Clinical Trial

**DOI:** 10.1001/jamanetworkopen.2023.50969

**Published:** 2024-01-16

**Authors:** Alison R. Carroll, Jakobi A. Johnson, Justine C. Stassun, Robert A. Greevy, Amanda S. Mixon, Derek J. Williams

**Affiliations:** 1 Division of Pediatric Hospital Medicine, Department of Pediatrics, Monroe Carell Jr Children’s Hospital at Vanderbilt, Vanderbilt University School of Medicine, Nashville, Tennessee; 2 Department of Biostatistics, Vanderbilt University School of Medicine, Nashville, Tennessee; 3 Section of Hospital Medicine, Division of General Internal Medicine and Public Health, Department of Internal Medicine, Vanderbilt University School of Medicine, Nashville, Tennessee

## Abstract

**Importance:**

Inadequate communication between caregivers and clinicians at hospital discharge contributes to medication dosing errors in children. Health literacy–informed communication strategies during medication counseling can reduce dosing errors but have not been tested in the pediatric hospital setting.

**Objective:**

To test a health literacy–informed communication intervention to decrease liquid medication dosing errors compared with standard counseling in hospitalized children.

**Design, Setting, and Participants:**

This parallel, randomized clinical trial was performed from June 22, 2021, to August 20, 2022, at a tertiary care, US children’s hospital. English- and Spanish-speaking caregivers of hospitalized children 6 years or younger prescribed a new, scheduled liquid medication at discharge were included in the analysis.

**Interventions:**

Permuted block (n = 4) randomization (1:1) to a health literacy–informed discharge medication communication bundle (n = 99) compared with standard counseling (n = 99). A study team member delivered the intervention consisting of a written, pictogram-based medication instruction sheet, teach back (caregivers state information taught), and demonstration of dosing with show back (caregivers show how they would draw the liquid medication in the syringe).

**Main Outcome and Measures:**

Observed dosing errors, assessed using a caregiver-submitted photograph of their child’s medication-filled syringe and expressed as the percentage difference from the prescribed dose. Secondary outcomes included caregiver-reported medication knowledge. Outcome measurements were blinded to participant group assignment.

**Results:**

Among 198 caregivers randomized (mean [SD] age, 31.4 [6.5] years; 186 women [93.9%]; 36 [18.2%] Hispanic or Latino and 158 [79.8%] White), the primary outcome was available for 151 (76.3%). The observed mean (SD) percentage dosing error was 1.0% (2.2 percentage points) among the intervention group and 3.3% (5.1 percentage points) among the standard counseling group (absolute difference, 2.3 [95% CI, 1.0-3.6] percentage points; *P* < .001). Twenty-four of 79 caregivers in the intervention group (30.4%) measured an incorrect dose compared with 39 of 72 (54.2%) in the standard counseling group (*P* = .003). The intervention enhanced caregiver-reported medication knowledge compared with the standard counseling group for medication dose (71 of 76 [93.4%] vs 55 of 69 [79.7%]; *P* = .03), duration of administration (65 of 76 [85.5%] vs 49 of 69 [71.0%]; *P* = .04), and correct reporting of 2 or more medication adverse effects (60 of 76 [78.9%] vs 13 of 69 [18.8%]; *P* < .001). There were no differences in knowledge of medication name, indication, frequency, or storage.

**Conclusions and Relevance:**

A health literacy–informed discharge medication communication bundle reduced home liquid medication administration errors and enhanced caregiver medication knowledge compared with standard counseling. Routine use of these standardized strategies can promote patient safety following hospital discharge.

**Trial Registration:**

ClinicalTrials.gov Identifier: NCT05143047

## Key Points

**Question:**

Does a health literacy–informed communication intervention, compared with standard counseling, reduce discharge medication errors in hospitalized children of English- and Spanish-speaking caregivers?

**Findings:**

In this randomized clinical trial including 198 caregivers of hospitalized children, a health literacy–informed communication intervention (written, pictogram-based medication instruction sheet, liquid medication dosing demonstration, and structured teach back and show back) resulted in significantly fewer caregiver medication errors and more accurate caregiver medication knowledge compared with standard counseling.

**Meaning:**

Findings of this trial indicate that health literacy–informed communication tools improve the safety of the discharge medication counseling process and should be the standard of care to facilitate safe transitions from hospital discharge to home.

## Teaser

This randomized clinical trial assesses whether a health literacy–informed communication intervention reduces discharge medication dosing errors and enhances medication knowledge compared with standard discharge counseling for caregivers of hospitalized children.

## Introduction

Errors in pediatric medication dosing are common, with approximately 1 out-of-hospital medication error occurring every 8 minutes among children younger than 6 years.^1,2,3^ Liquid medications account for most pediatric dosing errors, likely due to inherent dosing complexities, including weight-based dosing, varying medication concentrations, and use of nonstandardized dosing instruments.^1,4^ The posthospitalization period may be an especially vulnerable time for medication errors. To date, however, most work on reducing pediatric out-of-hospital medication errors have focused on clinic or emergency department settings, and no randomized interventions, to our knowledge, have been investigated in the inpatient setting.^2,5,6,7,8^

Only 15% of parents possess proficient levels of health literacy, indicating that most are at risk for health literacy–related challenges.^9^ Limited health literacy has been associated with a 1.5- to 2.5-fold increased odds of liquid medication dosing errors.^6,10,11^ In 1 study conducted among hospitalized children,^12^ 70% of parents with suspected poor health literacy had a postdischarge medication-related error. The anxiety, stress, and poor sleep often experienced by caregivers during their child’s hospitalization may temporarily worsen health literacy and further interfere with caregivers’ ability to effectively manage discharge instructions.^13,14,15,16^

Health literacy–informed communication strategies—plain language, pictures or pictograms, teach back, and demonstration with show back—improve communication and comprehension and reduce medication errors and patient harm.^5,17,18,19,20,21,22^ However, clinicians infrequently use these strategies, citing barriers, such as time constraints and competing patient priorities.^15,23^ Few studies have evaluated the efficacy of these bundled communication strategies tailored to the unique pediatric inpatient setting. We tested the hypothesis that a health literacy–informed communication intervention would reduce discharge medication dosing errors and enhance caregiver medication knowledge compared with standard discharge counseling for hospitalized children.

## Methods

### Trial Design and Participants

**Figure 1.**
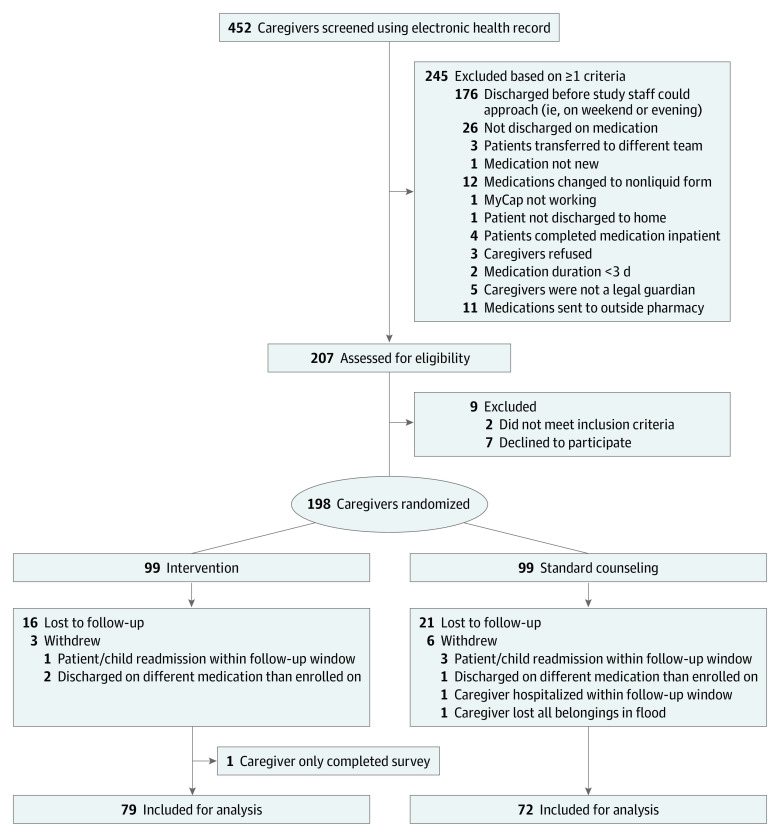
CONSORT Diagram of Enrollment and Randomization

We conducted a parallel group, randomized clinical trial from June 22, 2021, to August 20, 2022, at a single tertiary care US children’s hospital. Permuted block (n = 4) randomization^24^ assigned caregiver-child dyads to intervention or control groups in a 1:1 allocation (Figure 1). Approval was obtained from the Vanderbilt University Medical Center Institutional Review Board, and caregivers provided written informed consent. The study protocol is found in Supplement 1. This trial followed the Consolidated Standards of Reporting Trials (CONSORT) reporting guideline.

Caregivers of hospitalized children admitted to hospital medicine teams were screened for enrollment using the electronic health record (EHR; Epic Systems Corporation). Inclusion criteria were a English- or Spanish-speaking caregiver who was the legal guardian of a child 6 years or younger being discharged home with a new, scheduled liquid medication for administration duration of at least 3 days. For those discharged with more than 1 new liquid medication, 1 medication was selected at random. Exclusion criteria included medication administration by a home health nurse, children in state custody, and discharge medications not filled at the hospital’s outpatient pharmacy. Eligible caregivers were approached by study staff prior to their child’s discharge on weekdays. Following consent, caregivers were randomized to standard discharge medication counseling or the intervention.

### Intervention

The intervention was a communication bundle developed using the best available evidence^5,10,13,18,20^ and previously completed qualitative interviews.^15^ The intervention included a patient-specific, plain language, pictogram-based written medication instruction sheet (eFigure in Supplement 2) consisting of a picture of the appropriately sized oral dosing syringe corresponding to the prescribed dose; a customized table with the medication name, indication, dose, frequency, duration, timing for next dose following discharge, and most common adverse effects (eAppendix in Supplement 2); and medication storage instructions. The written instruction sheet was manually customized with the patient-specific medication information by the study principal investigator (PI) (A.R.C). The study PI, a pediatric hospitalist (A.R.C.), or a trained research assistant (J.A.J.) delivered the intervention on the day of discharge.

Study personnel reviewed all aspects of the written medication instruction sheet with caregivers and assessed caregiver comprehension using the teach-back method (caregivers state information taught to them in their own words).^25^ Study staff then demonstrated how to measure the patient’s dose using an oral dosing syringe. Caregivers were asked to show back this technique. Additional teaching was provided if needed. A standardized, graduated dosing syringe (1, 3, 5, 10, or 20 mL) was provided to all caregivers. Caregivers also received standard discharge medication counseling per hospital policy.

### Standard Medication Counseling

Caregivers in the standard medication counseling group received standard of care discharge communication from pediatric nursing staff regarding prescribed medications, postdischarge instructions, return precautions, and follow-up appointments. Written discharge instructions were created within the EHR.

### Baseline Characteristics

Baseline characteristics were assessed on the day of discharge, and outcomes were assessed 48 to 72 hours later. Study data were collected and managed using REDCap (Research Electronic Data Capture) hosted at Vanderbilt University.^26,27^ Self-reported caregiver characteristics included age, sex, relationship to patient, race and ethnicity, preferred language, birth country, educational level, and difficulty paying bills (proxy for financial stress). Child information included age, sex, race and ethnicity, number of daily home medications, and number of previous hospitalizations in the last year. Race and ethnicity were collected according to funding agency requirements.

Caregiver health literacy was assessed using the Newest Vital Sign (NVS), a 6-item screening tool that assesses an individual’s numeracy, prose, and document literacy while referencing a food nutrition label.^28^ Scores on the NVS range from 0 to 6, with higher scores indicating adequate health literacy. The validated NVS has been used in multiple clinical studies to assess health literacy.^29,30,31^ Additional demographic and clinical information was abstracted from the EHR: child’s insurance, number of complex chronic conditions,^32^ and medication prescription details.

### Primary and Secondary Outcomes

The primary outcome was observed dosing error, which was assessed using a caregiver-submitted photograph of their child’s medication-filled syringe and expressed as the continuous percentage difference from the prescribed dose in the EHR. We chose a continuous measure of dosing error because no standardized, clinically meaningful definition of dosing error exists, and this approach best captures the full spectrum of possible dosing errors. Outcomes were collected using MyCap (Vanderbilt University), a Health Insurance Portability and Accountability Act–compliant, participant-facing mobile application for survey data collection and automated administration of study tasks, including transmission of high-resolution images.^33^ After enrollment, study staff assisted caregivers in downloading MyCap on their mobile devices and demonstrated how to complete the image upload and follow-up survey. An automated MyCap push notification reminded caregivers to complete follow-up tasks 48 hours following discharge. Study staff also sent text, telephone, and email reminders. Caregivers in the intervention group were encouraged to use their instruction sheet when drawing up the dose and answering the follow-up survey questions. All caregivers were asked to draw up a single dose of their child’s medication using the provided oral syringe and send a picture of the medication-filled syringe via MyCap. The observed measured dose was estimated via visual inspection by 2 trained study staff (J.A.J. and J.C.S.) who were blinded to group assignment. Reviewers agreed on the exact dose (to the nearest 0.1 mL or 0.01 mL, depending on the syringe size) in 93% of the cases. Discrepancies were resolved by the study PI (A.R.C).

Caregiver self-reported secondary outcomes were collected via a MyCap survey, including knowledge of medication name, dose, indication, frequency, duration, storage, and adverse effects. Knowledge of adverse effects was categorized as 0, 1, or 2 or more correctly identified using an established list of each medication’s most common adverse effects,^12^ validated using Lexicomp,^34^ and reviewed by a pediatric clinical pharmacist (eAppendix in Supplement 2). All other responses were dichotomized as correct or incorrect based on the medication prescription.

### Statistical Analysis

We estimated needing a sample size of 72 caregivers in each arm (144 total) to reject the null hypothesis that the dosing error prevalence in the control and intervention arms was equal with 80% power (2-sided α = .05) assuming a dosing error prevalence of 30% in the standard counseling group.^5,8,35,36,37^ Accounting for 10% loss to follow-up, we planned to enroll 160 caregivers.

Statistical analyses were performed using Stata, version 16.1 (StataCorp LLC). The primary analysis followed intention-to-treat principles and included all caregivers who were randomized and completed the MyCap picture upload of their child’s medication dose. Means and SDs for the primary outcome of percentage difference in observed dosing were compared between groups using a 2-tailed *t* test. Secondary outcomes were assessed using 2-sided Fisher exact tests. Two-sided *P* < .05 indicated statistical significance. We planned a secondary analysis a priori to control for potential chance imbalances in baseline characteristics using propensity score adjustment via inverse probability of treatment weighting. The eMethods and eTable 1 in Supplement 2 provide further details. In an exploratory post hoc analysis, the cohort was also stratified by health literacy level (ie, limited vs adequate health literacy score) given the established relationship between health literacy and medication dosing errors.^2,5,17,20,22^

## Results

### Participants

Among 452 screened caregivers, 207 were approached to confirm eligibility, and 198 were enrolled and randomized (mean [SD] age, 31.4 [6.5] years; 186 women [93.9%] and 12 men [6.1%]) (Figure 1). In terms of race and ethnicity, 1 caregiver (0.5%) was Asian; 28 (14.1%), Black; 36 (18.2%), Hispanic or Latino; 3 (1.5%), Native Hawaiian or Other Pacific Islander; 158 (79.8%), White; and 3 (1.5%), multiracial. Five caregivers (2.5%) preferred not to answer (Table 1).

**Table 1.** Characteristics of Enrolled Children and Caregivers by Study Group (n = 198)

Characteristic	Study group participants, No (%)^a^	
	Intervention (n = 99)	Standard counseling (n = 99)
**Children**		
Age, mean (SD), y	2.4 (2.1)	2.4 (2.0)
Sex		
Boys	56 (56.6)	54 (54.5)
Girls	43 (43.4)	45 (44.5)
Race^b^		
Asian	1 (1.0)	0
Black or African American	10 (10.1)	16 (16.2)
Native Hawaiian/Pacific Islander	3 (3.0)	0
White	75 (75.8)	67 (67.7)
Multiracial	9 (9.0)	12 (12.1)
Prefer not to answer	1 (1.0)	3 (3.0)
Hispanic or Latino ethnicity	19 (19.2)	25 (25.3)
Uses medication(s) regularly^c^	26 (26.3)	24 (24.2)
≥2 Hospitalizations in last year	14 (14.1)	19 (19.2)
Complex chronic condition present	27 (27.3)	20 (20.2)
Payer		
Government	52 (52.5)	72 (72.7)
Private	46 (46.5)	26 (26.3)
No insurance	1 (1.0)	0
Other	0	1 (1.0)
Discharge medication type		
Antimicrobials	84 (84.8)	85 (85.9)
Anticonvulsants	2 (2.0)	2 (2.0)
Corticosteroids	2 (2.0)	4 (4.0)
Gastrointestinal agents/antacids	4 (4.0)	3 (3.0)
Other^d^	6 (6.1)	5 (5.1)
**Caregivers**		
Age, mean (SD), y	32.1 (7.0)	30.6 (5.9)
Sex		
Men	7 (7.1)	5 (5.1)
Women	92 (92.9)	94 (94.9)
Relationship to child		
Mother	91 (91.9)	91 (91.9)
Father	7 (7.1)	7 (7.1)
Grandparent	0	1 (1.0)
Legal guardian	1 (1.0)	0
Race		
Asian	1 (1.0)	0
Black or African American	13 (13.1)	15 (15.2)
Native Hawaiian or Other Pacific Islander	3 (3.0)	0
White	80 (80.8)	78 (78.8)
Multiracial	1 (1.0)	2 (2.0)
Prefer not to answer	1 (1.0)	4 (4.0)
Hispanic or Latino ethnicity	12 (12.1)	24 (24.2)
Spanish speaking	8 (8.1)	19 (19.2)
Born outside the US	14 (14.1)	26 (26.3)
Educational level		
Less than high school diploma	14 (14.1)	16 (16.2)
High school degree or equivalent	23 (23.2)	31 (31.3)
Some college, no degree	30 (30.3)	28 (28.3)
College degree	22 (22.2)	16 (16.2)
Graduate degree	10 (10.1)	8 (8.1)
Difficulty paying bills at home	14 (14.1)	21 (21.2)
Newest Vital Sign health literacy score		
Low (0-1)	11 (11.1)	15 (15.2)
Marginal (2-3)	31 (31.3)	42 (42.4)
Adequate (4-6)	57 (57.6)	42 (42.4)

^a^ Percentages may not sum to 100% owing to rounding.

^b^ Data were missing for 1 child in the standard counseling group.

^c^ Indicates 1 or more daily scheduled medications at home.

^d^ Include cardiovascular agents and vitamins.

Of the 198 randomized caregivers, 151 (76.3%) completed the primary outcome assessment of observed dosing error (79 in the intervention group and 72 in the standard counseling group), and 145 (73.2%) completed the survey of medication knowledge (76 in the intervention group and 69 in the standard counseling group). There were no differences in baseline characteristics between those who did and did not complete the primary outcome assessment (eTable 2 in Supplement 2).

Mean (SD) caregiver age was similar across groups (intervention: 32.1 [7.0] years; standard counseling: 30.6 [5.9] years). Between-group demographic differences included caregiver ethnicity and language, birth outside the US, and insurance payer (Table 1). The mean (SD) health literacy score was 3.1 (1.6) in the standard counseling group compared with 3.7 (1.8) in the intervention group (mean difference, 0.61 [95% CI, 0.13-1.10]; *P* = .01). Most discharge medications (for 84 [84.8%] in the intervention group and 85 [85.9%] in the standard counseling group) were antimicrobials (Table 1). The mean (SD) intervention delivery time was 5 minutes and 9 seconds (4 minutes and 39 seconds).

### Observed Dosing Error

**Figure 2.**
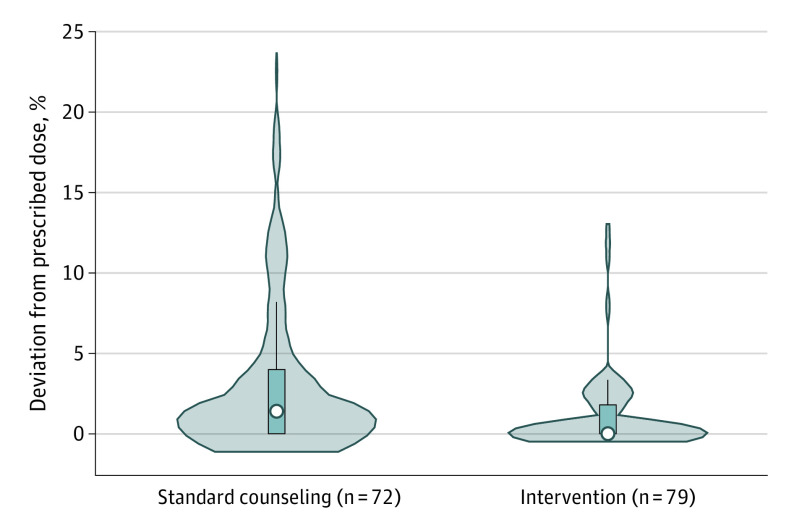
Observed Dosing Error Among Caregivers of Children Discharged With a New, Scheduled Liquid Medication White dot for standard counseling indicates a median of 1.4%; dark blue box, IQR of 0%-4.0%. White dot for intervention indicates a median of 0%; dark blue box, IQR of 0%-1.8%. Thin blue line represents the rest of the distribution, and the widest portion of each plot represents the most frequent outcome. A Wilcoxon rank-sum test of the median percentage deviation from the prescribed dose between the standard counseling group and the intervention group was significant at *P* < .001.

Sixty-three of 151 caregivers (41.7%) had dosing errors, with significantly more errors in the standard counseling group compared with the intervention group (39 of 72 [54.2%] vs 24 of 79 [30.4%]; *P* = .003). The range of percentage difference in dosing errors was 0 to 22.4% in the standard counseling group vs 0 to 12.5% in the intervention group (Figure 2). The unweighted mean (SD) observed dosing error was 1.0% (2.2 percentage points) among the intervention group and 3.3% (5.1 percentage points) among the standard counseling group (absolute difference, 2.3 [95% CI, 1.0-3.6] percentage points; *P* < .001). The inverse probability of treatment weighting adjustment balanced all major covariates (eTable 1 in Supplement 2), and the weighted mean difference in observed percentage dosing error was 1.5 percentage points (95% CI, 0.1-2.9 percentage points; *P* = .04). Violin plots (Figure 2) demonstrate the variability in dosing errors between the 2 groups. Only 2 caregivers (2.5%) in the intervention group had more than 10% deviation from the prescribed dose compared with 9 caregivers (12.5%) in the standard counseling group (*P* = .02), corresponding to a number needed to treat of 10 participants to avoid 1 dosing error of 10% or greater from prescribed dose (95% CI, 5.0-68.3 participants; *P* = .02).

In our exploratory post hoc analysis stratified by health literacy level (ie, limited vs adequate health literacy), the mean (SD) magnitude of dosing errors was generally higher among those with limited health literacy (1.7% [3.3%] among intervention group caregivers [n = 30] and 3.8% [5.5%] among standard counseling group caregivers [n = 41]) compared with those with adequate health literacy (0.6% [1.1%] among intervention group caregivers [n = 49] and 2.7% [4.5%] among standard counseling caregivers [n = 31]). However, the absolute percentage difference in observed dosing error between the intervention and standard counseling groups in those with limited health literacy (2.10% [95% CI, −0.15% to 4.40%]; *P* = .07) was similar to the absolute difference between the intervention and standard counseling groups in those with adequate health literacy (2.10% [95% CI, 0.74%-3.40%; *P* = .003).

### Caregiver Medication Knowledge

When caregivers were asked to report the dose of their child’s medication (to the nearest 0.1 mL or 0.01 mL, depending on the syringe) on the follow-up survey, the reported mean (SD) dosing error was 0.4% (3.0%) among the intervention group and 2.0% (6.0%) in the standard counseling group (absolute difference, 1.60 [95% CI, 0.05-3.10] percentage points; *P* = .04) (Table 2). Caregivers in the intervention group also had greater knowledge of medication dose (71 [93.4%] vs 55 [79.7%]; *P* = .03) and duration (65 [85.5%] vs 49 [71.0%]; *P* = .04) and more correct reporting of 2 or more adverse effects of medication (60 [78.9%] vs 13 [18.8%]; *P* < .001). There were no differences in knowledge of medication name (70 [92.1%] vs 60 [87.0%]; *P* = .42), indication (71 [93.4%] vs 59 [85.5%]; *P* = .17), frequency (74 [97.4%] vs 65 [94.2%]; *P* = .42), or storage (76 [100%] vs 67 [97.1%]; *P* = .22).

**Table 2.** Primary and Secondary Study Outcomes

Outcome	Study group		*P* value
	Intervention (n = 76)	Standard counseling (n = 69)	
Difference in observed medication dose, mean (SD), %^a^	1.0 (2.2)	3.3 (5.1)	<.001^b^
Difference in reported medication dose, mean (SD), %^c^	0.4 (3.0)	2.0 (6.0)	.04^b^
Correct medication name^d^	70 (92.1)	60 (87.0)	.42^e^
Correct medication indication^f^	71 (93.4)	59 (85.5)	.17^e^
Correct medication dose^g^	71 (93.4)	55 (79.7)	.03^e^
Correct medication frequency^h^	74 (97.4)	65 (94.2)	.42^e^
Correct medication duration^i^	65 (85.6)	49 (71.0)	.04^e^
Correct medication adverse effects reporting^j^	60 (78.9)	13 (18.8)	<.001^e^
Correct medication storage^k^	76 (100)	67 (97.1)	.22^e^

^a^ Calculated by comparing dose in caregiver submitted photograph of oral dosing syringe compared with dose prescribed in electronic health record. Includes 79 for intervention group and 72 for standard counseling group.

^b^ Calculated using a 2-tailed *t* test.

^c^ Includes 75 for intervention group and 68 for standard counseling group. Calculated by comparing the dose of medication reported in caregiver follow-up survey compared with dose prescribed in the electronic health record.

^d^ Calculated as any error in medication name comparing caregiver report on follow-up survey and the electronic health record.

^e^ Calculated using Fisher exact test.

^f^ Calculated as any error in medication indication comparing caregiver report on follow-up survey and the electronic health record.

^g^ Calculated as any error in medication dose comparing caregiver report on follow-up survey and the electronic health record.

^h^ Calculated as any error in medication frequency comparing caregiver report on follow-up survey and the electronic health record.

^i^ Calculated as any error in medication duration comparing caregiver report on follow-up survey and the electronic health record.

^j^ Calculated as number and percent of caregivers who reported 2 or more correct medication adverse effects compared with the established list (eAppendix in Supplement 2).

^k^ Calculated as any deviation in medication storage comparing caregiver report on follow-up survey and the electronic health record.

## Discussion

In this single center, randomized clinical trial, a health literacy–informed discharge medication communication bundle compared with standard counseling resulted in fewer liquid medication dosing errors and enhanced medication knowledge among caregivers of children following hospital discharge. The brief and low-resource nature of the intervention, coupled with the observed efficacy, supports universal application of health literacy–informed discharge communication strategies to improve care delivery and outcomes for hospitalized children.

Our findings are consistent with previous work in the pediatric outpatient setting and pediatric ED demonstrating that 40% to 50% of caregivers make errors in administering medications to children^36,37,38^ and that health literacy–informed communication strategies are efficacious in reducing dosing errors.^6,11^ A randomized clinical trial in a single-center, pediatric ED that used a similar communication intervention bundle^5^ found that nearly half of caregivers gave a dose of medication that deviated more than 20% from what was prescribed. While the magnitude of dosing deviation error in our study was not as large in comparison, this is likely due to differences in definitions of dosing error, the method for assessing dosing error (pictures vs in-person), follow-up time for outcome assessment (2-3 days in our study vs 12 days), and overall increased parental awareness of medication safety.^25^ Given that there is not a standard definition for clinically meaningful dosing errors, we used a continuous percentage deviation from prescribed dose to best capture the full spectrum of potential errors. We posit that capturing images of doses measured in the home by the caregiver via MyCap is a more pragmatic and patient-centric measure that better mirrors clinical conditions where even small dosing errors have the potential for adverse events, depending on patient- and medication-specific factors.

Using our definition of dosing error, the percentage of caregivers who made any error was nearly halved from 54.2% in the standard counseling group to 30.4% in the intervention group. While the mean magnitude of dosing error was small (2.3%) across both groups, persistent errors across multiple doses increase the risk for harm, particularly for medications with narrow therapeutic windows or among children receiving multiple medications. While the medications included in this study were primarily antimicrobials, which do not typically have a narrow therapeutic index, incorrect medication dosing by even a small amount can be detrimental for children with chronic conditions and/or medical complexity (nearly one-quarter of enrolled children in our study) who are often prescribed multiple medications or medications that have complex instructions.^39,40,41,42,43^ Our finding of significantly fewer errors at greater than 10% deviation from the prescribed dose in the intervention group suggests that the intervention likely mitigated the most dangerous errors: extreme overdosing or underdosing of medications. Significantly fewer caregivers in the intervention made any error, supporting the goal of eliminating home medication administration errors for all children. Furthermore, the intervention is simple, quick, and effective for caregivers of all literacy levels, suggesting it could be an effective and feasible intervention for implementation across many hospitals.

Low parent health literacy is associated with medication dosing and adherence errors.^5,7,38,44,45^ In our analyses stratified by health literacy, there were greater dosing errors among caregivers with low health literacy in both arms compared with caregivers with adequate health literacy. However, the magnitude of benefit conferred by the intervention was similar in both groups. This suggests the intervention is likely useful for all caregivers regardless of health literacy, while also underscoring the vulnerability of those with lower health literacy to medication dosing errors. These results support using universal precautions for health literacy to minimize dosing errors and promote equity. Examining the efficacy of tailored interventions in different subpopulations, including varying levels of health literacy, education, or primary language, could be informative.

Our intervention also improved caregiver medication knowledge for dose, duration, and adverse effects. Yin et al^5^ similarly found a significant difference in caregiver knowledge of medication dose but did not assess knowledge of duration or adverse effects. In our study, we found no differences for knowledge of medication name, indication, or storage, consistent with previous work.^5^ This may indicate that these aspects of medication counseling are easier for caregivers to understand and/or reference (ie, on a medication bottle label). Regardless, errors in knowledge lead to errors in medication administration.^46^ When examining discharge instruction comprehension and adherence errors for hospitalized children, Glick et al^47^ found that parents who made comprehension errors had approximately 9 times the odds of making adherence errors, underscoring the importance of confirming caregiver understanding using teach-back and show-back methods prior to hospital discharge.

Caregiver understanding of the adverse effects of medication is understudied. In 1 study,^48^ over 40% of parents overestimated their comprehension of potential adverse effects of medication. In our study, only 18.8% of caregivers in the standard counseling group correctly reported 2 or more adverse effects of medication compared with 78.9% in the intervention group. Unfamiliarity with adverse effects could lead caregivers to seek care for benign or expected adverse effects of medication (ie, diarrhea from an antibiotic) or delay recognition of serious adverse effects. Review of the adverse effects of medication is an often overlooked but critical component of hospital discharge counseling for children.

### Limitations

Important study limitations should be considered. First, we only included English- and Spanish-speaking caregivers recruited from a single institution. It is possible that the intervention might have had differential effects in other populations of caregivers or other institutions. Second, the rate of loss to follow-up was higher than anticipated. Baseline characteristics between those completing the study and those lost to follow-up were similar (eTable 2 in Supplement 2), and the rate of loss to follow-up was similar between groups. Therefore, it is unlikely that the observed loss to follow-up would materially change our conclusions. Third, our design may have limited the magnitude of dosing errors by providing caregivers with the smallest, most appropriate oral dosing syringe. While necessary for safety, this placed an upper bound on dosing errors. Finally, the follow-up duration for our study was 2 to 3 days, and caregiver retention of medication teaching may vary based on follow-up timing.

## Conclusions

In this randomized clinical trial, a simple, brief communication intervention for discharge medication counseling can reduce medication dosing errors in hospitalized children once they return home. Future work should focus on adapting the existing EHR discharge medication list or using existing EHR-embedded tools to generate patient-specific, pictogram-based medication instruction sheets. In addition, all clinicians who participate in discharge counseling should be trained to consistently use teach-back and show-back methods. A future pragmatic trial assessing effectiveness and measuring implementation outcomes would be helpful in testing EHR adaptations.

## Notes

Supplement 1.
Trial Protocol

Supplement 2. **eFigure.** Example of Written, Pictogram-Based Medication Instruction Sheet **eAppendix.** Medication Indications, Adverse Effects, and Storage Information **eMethods.** Inverse Probability of Treatment Weighting Analysis **eTable 1.** Inverse Propensity Score Weighting of Characteristics of Children and Caregivers by Randomization Group for Primary Outcome (n=151) **eTable 2.** Comparison of Participants Included in Analysis and Those Lost to Follow-up or Withdrawn

Supplement 3.
Data Sharing Statement
