# Supplementary material for: Health Literacy–Informed Communication to Reduce Discharge Medication Errors in Hospitalized Children: A Randomized Clinical Trial
Source: JAMA Netw Open. 2024 Jan 16;7(1):e2350969. doi: 10.1001/jamanetworkopen.2023.50969 (PMC10792470; doi:10.1001/jamanetworkopen.2023.50969)
Supplement: Supplement 1. — Trial Protocol [file jamanetwopen-e2350969-s001.pdf]

Development and Evaluation of a Health Literacy-Informed Communication Intervention for Discharge Medication Counseling in Hospitalized Children

Principal Investigator
Alison R. Carroll, MD
Pediatric Hospital Medicine Fellow
Department of Pediatrics
Monroe Carrel Jr. Children's Hospital at Vanderbilt
Vanderbilt University Medication Center

Co-Investigators
Derek J. Williams, MD MPH
Amanda Mixon, MD, MSPH

Last Revision: 4/21/2022

Table of Contents:

1.0 Background
2.0 Rationale and Specific Aim
3.0 Study Design, Study Population, Statistical Analysis and Sample Size Calculation
4.0 Risk to Participants
5.0 Privacy/Confidentiality Issues
6.0 Reporting of Adverse Events or Unanticipated Problems Involving Risk to Participants
7.0 References

Protocol Summary

Caregivers of children age ≤ 6 years old who speak English or Spanish and are discharged home from the inpatient hospital medicine team at Monroe Carell Jr. Children's Hospital at Vanderbilt on a new liquid medication will be potentially eligible for the study. Caregivers will be randomized at the time of enrollment to receive usual care for discharge medication teaching or receive enhanced discharge medication teaching which is a health literacy-informed communication intervention including a written medication sheet, teach-back, provision of an oral dosing syringe and show-back of proper liquid medication administration. Caregivers will be enrolled in person prior to their child's discharge home from the hospital. A follow-up survey conducted via myCap will be completed 48 to 72 hours following discharge to assess study outcomes. The anticipated study duration is 2 years total of which 12 months is anticipated for subject recruitment and enrollment starting July 1, 2021.

1.0 Background

One out-of-hospital medication error occurs every 8 minutes among children < 6 years of age.[1] Medication errors are particularly common in infants and young children, likely due to the near universal use of liquid medications in this population. As compared to pills or tablets, oral liquid medications present unique challenges (e.g., varying concentrations, dose measurement, and the frequent use of non-standardized dosing instruments) and account for the majority of pediatric dosing errors. Prior studies estimate that liquid medication dosing errors in children are very common with over 50% of caregivers measuring an incorrect dose or reporting giving a dose outside of the recommended range.[2–6] Poor medication adherence (e.g. missed doses) further compounds the problem of medication errors in children with potential implications for treatment failure and, in the case of antibiotics, development of drug resistance.[7,8]

More than 1.5 million children are hospitalized in the United States each year, and the transition from hospital to home is a vulnerable time for medication errors.[9] Most children receive multiple medications during a hospital stay.[10] Many of these medications are new and many are continued at home. This raises the risk for errors as the responsibility of medication administration shifts from trained health care providers to caregivers, many of whom experience increased stress and exhaustion during a hospitalization making comprehension of medication instructions more difficult.

Health literacy, or the ability to understand health information to make appropriate health decisions, is a key driver of effective communication, especially when discussing medication administration.[11] Low health literacy is common, present in 29% of US parents, and is associated with increased risk of medication errors.[11] It is thus imperative to consider health literacy as a critical element of effective communication with caregivers in the transition from hospital to home in order to minimize medication errors. Several studies in outpatients have demonstrated the usefulness of health literacy-informed communication strategies (written instructions, teach-back, show-back) for reducing medication errors.[12–14] Evaluations of health literacy-informed communication interventions calibrated to the unique complexities and needs of caregivers in the transition from hospital to home, however, are lacking.

2.0 Rationale and Specific Aim

Despite the association of poor health literacy of caregivers with worse health outcomes in children, actual caregiver health literacy has been assessed in only 5 emergency department studies and no inpatient studies which examined medication errors.[13,15–18] Addressing health literacy is integral to any proposed solution seeking to eliminate out-of-hospital medication errors as children transition from the hospital to home. Given the exhaustion and stress of a hospitalization, all caregivers are at risk to perform below their normal level of health literacy. Therefore, healthy literacy "universal precautions" should be followed by all health care workers which means assuming that all patients and caregivers may have difficulty comprehending health information.[19] The Joint Commission has stated that unaddressed health literacy issues undermines the safety of patients, and health care organizations must identify and meet patient's oral and written communication needs.[20] Ways to meet these needs and improve medication safety include using proven communication strategies, such as teach-back, show-back, and providing written information.[21,22] Use of pictograms along with

written instructions is associated with improved comprehension and adherence with medical instructions, especially in those with low health literacy.[2,14,23,24] Unfortunately, standardization and utilization of proven communication strategies in the hospital is often poor.[21,25]

Prior work has highlighted the commonality of liquid medication errors in infants and young children and the potential of enhanced communication strategies to reduce errors, resulting in care that is safer and more effective. However, few studies have examined health literacy-informed interventions to decrease discharge liquid medication administration errors and improve medication adherence in hospitalized children during the transition from hospital to home. Our proposed study directly addresses this important knowledge gap.

The overarching goal of this research is to make post-discharge care for children safer and more effective. Our objective is to 1) design and test the efficacy of a health literacy-informed discharge medication counseling intervention in the inpatient setting to reduce medication dosing errors by caregivers and improve adherence in infants and young children discharged home on a liquid medication. The specific aim of this study is:

Specific Aim In a randomized controlled trial, we will test the efficacy of a health literacy-informed communication intervention on caregiver discharge medication dosing accuracy, adherence, and comprehension. *We hypothesize that a useable, health literacy-informed medication counseling intervention will be efficacious in reducing liquid medication dosing errors and improve adherence and medication comprehension among caregivers following hospital discharge for children receiving liquid medication as compared to usual care.*

The primary outcome is observed dosing accuracy assessed via picture of an oral dosing syringe submitted by a caregiver 48-72 hours following discharge and compared to the prescribed dose in the chart. The primary outcome will be a continuous measure of percent difference of the caregiver submitted photo compared to the prescribed dose (in mL). The primary measure will also be assessed as a dichotomous variable as +/- 20% from the prescribed dose. Secondary outcomes include caregiver medication adherence and medication comprehension assessed via caregiver follow-up survey.

3.0 Study Design, Study Population, Statistical Analysis and Sample Size Calculation

3.1 Study Design and Study Population

We will conduct a randomized controlled trial (RCT) over a period of 12 months among English- and Spanish-speaking caregivers of children age birth to ≤ 6 years being discharged from the hospital following an acute care inpatient stay in which at least one new liquid medication is initiated to be continued at home. Inclusion and exclusion criteria are outlined in Table 2. In addition, we will only include caregivers who will be or have already obtained their child's medicine from the Vanderbilt Children's Outpatient Pharmacy and are willing to download and use an application on their phone called myCap to complete the follow-up survey.

Table 2. RCT inclusion and exclusion criteria

Inclusion Criteria	Exclusion Criteria
Caregivers of children from birth to < 4 years	Medications administrated by home health nurse
English or Spanish speaking	Caregiver not fluent in English or Spanish
Discharged on a new scheduled liquid medication for a minimum duration of at least 3 days	Child in state custody (i.e., Department of Child Services)

3.2 Screening and Enrollment

Caregivers will be screened for eligibility Monday through Friday from 7 am to 6 pm. The PI or trained research assistant(s) will review currently admitted patients on the general hospital medicine teams who meet eligibility criteria and contact the attending physician to inquire about discharge status and request permission to approach the caregiver. For patients nearing discharge, caregivers will be approached to discuss the study and confirm eligibility (see Interest in Participation Script). An eligibility script will be used to standardize this process (see included document) Patient-level randomization will occur at the time of enrollment. Allocation to intervention or usual care will done by block randomization and will be assigned when individuals are determined to be eligible based on the eligibility script. Due to the nature of the intervention, it will not be possible to blind study staff or caregivers to the assigned study arm (intervention or usual care).

At the time of enrollment (i.e., either the day prior to discharge, if possible, or the day of discharge), caregivers in both groups will complete a demographics questionnaire via a secure web platform called REDCap (Research Electronic Data Capture).[26] Questions will include patient (child) age, sex, race/ethnicity, whether they were receiving any liquid medication prior to admission, number of previous hospitalizations, and number of previous daily medications. Demographic questions about caregivers will include age, gender, race/ethnicity, education level, caregiver relationship to patient, marital status of caregiver, education level of caregiver, employment status, difficulties paying bills (proxy for financial stress), country of origin, and preferred language. The following information will be extracted from the electronic health record: child discharge diagnosis, medication(s) name, number, frequency, dose and duration of discharge medication(s), patient insurance status, and number of chronic conditions.[27] Caregiver health literacy will be measured using the Newest Vital Sign (see included document).[28] The Newest Vital Sign has been translated into Spanish by the creators. See Figure 1 for study flow.

Figure 1. Aim 2 Study Flow

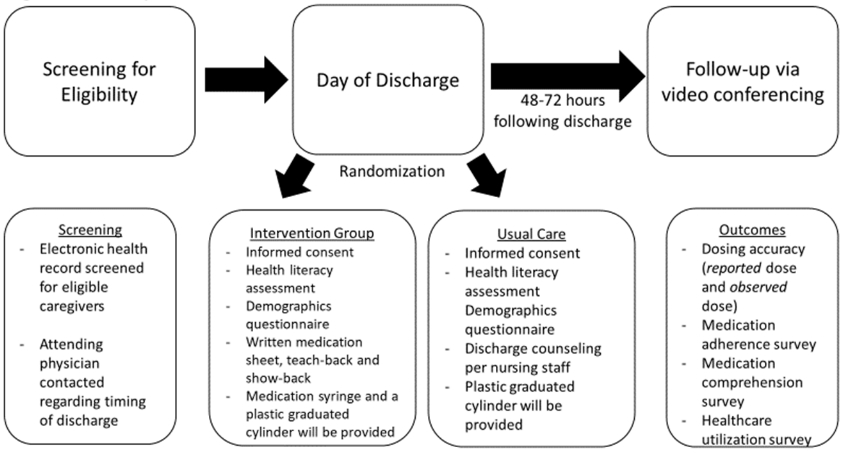

3.3 Intervention

Intervention: The design of the medication instruction sheet was guided by previous research, our previous qualitative study (IRB#201452), and work by Yin et al.[15] The medication instruction sheet will include a pictogram of a syringe marked with the correct dose of medication as well as information about the dose frequency, route of administration, length of treatment, storage and most common medication side effects (see example medication instruction sheet). The medication instruction sheets will be customized for each patient based on the discharge medicine, printed and used to facilitate discharge medication counseling which will include demonstration of accurate dosing with a standardized instrument (i.e., dosing syringe) by research staff followed by caregiver "show-back" of how they plan to administer the medication at home and "teach-back" regarding dosing amounts, times and end dates of medications and common side effects. This will be standardized within an intervention script (see included document) and will be piloted prior to implementation. A clear dosing syringe will be provided to the family prior to discharge. The syringe will be the smallest syringe that would allow administration of the appropriate dose. Caregivers will be given the instruction sheet to use at home. If children receive more than one liquid discharge medication, the intervention will be completed for only one randomly selected medication. We anticipate the intervention will take approximately 10 minutes. We anticipate that the eligibility screening, initial survey and intervention will take approximately 25 minutes to complete.

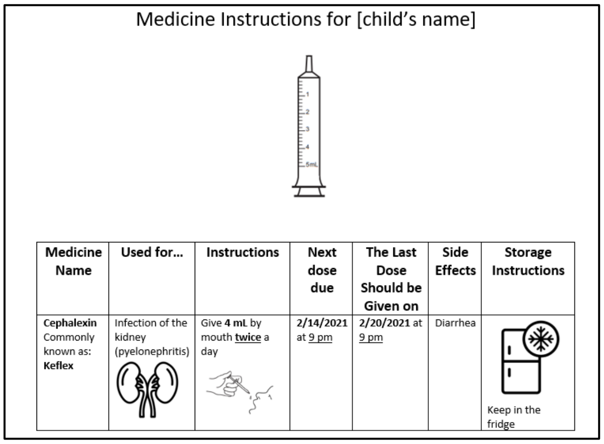
Medicine Instructions for [child's name]

Medicine Name	Used for…	Instructions	Next dose due	The Last Dose Should be Given on	Side Effects	Storage Instructions
**Cephalexin** Commonly known as: **Keflex**	Infection of the kidney (pyelonephritis)	Give **4 mL** by mouth **twice** a day	**2/14/2021** at 9 pm	**2/20/2021** at 9 pm	Diarrhea	Keep in the fridge

Usual Care: Caregivers in the usual care group will receive standard of care discharge communications per unit routine, including counseling regarding prescribed medications and post-discharge instructions, return precautions, and follow-up appointments by the pediatric nursing staff. At our institution, children discharged with a liquid medication from our outpatient pharmacy are provided with a dosing syringe as part of routine care. All communications about discharge medication instructions will be at the discretion of the clinical team as part of routine care. An additional dosing syringe will be supplied in a sealed envelope to be used at the discharge follow-up visit in order to standardize the assessment of the primary outcome of observed dosing accuracy.

3.4 Assessment of Outcomes

Follow-up Survey via myCap: The follow-up survey will be completed 48 to 72 hours following discharge to assess study outcomes. This will be done via myCap. myCap is an application that research participants will be asked to download on their phones (application is free and available on iPhone and Android phones). myCap leverages REDCap to capture participant/patient reported outcomes via mobile devices. myCap is HIPPA-compliant and secure. All data collected via myCap converts results into a format compatible with REDCap. The primary outcome of dosing accuracy will be assessed along with secondary outcomes (medication adherence and medication comprehension) via surveys in myCap. Caregivers will also be asked who administered the medication, what kind of teaching they received in the hospital prior to discharged (i.e., did teach-back, show-back occur), whether any serious medication errors occurred with the patient or other children in the home and/or if there were unplanned visits to a health care provider (see included follow-up survey). Caregiver self-efficacy for administering medicines to their children will be assessed using a standardized scale called the Medication Understanding and Use Self-Efficacy Scale (MUSE). See included document for RCT follow-up survey. We anticipate that the caregiver follow-up survey this will take <15 minutes to complete.

Primary Outcome: Dosing accuracy will be the primary outcome and will be assessed through self-report (reported dose) and through observation (observed dose) at follow-up. The primary outcome will be *observed dosing accuracy*. For *observed dosing accuracy,* caregivers will be asked to draw up a dose of their child's medication using the provided oral dosing syringe and send a secure picture of the syringe via myCap to research study staff (see Figure 2 as an example). The primary outcome will be assessed as a continuous percent difference from the prescribed dose (in milliliters). For *reported dose*, caregivers will be asked to report the prescribed volume (in milliliters) of medication to be given with each dose during the follow-up myCap survey. This will be compared to the prescribed dose in the electronic medical record. For both measures, dosing accuracy will also be assessed as a dichotomous outcome and considered accurate if it is within 20% of the prescribed dose similar to prior studies.[5,15]

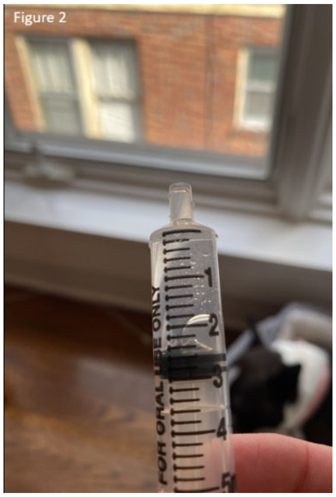
Figure 2

Safety Monitoring Plan: If any dosing errors are detected via the myCap picture and/or myCap follow-up survey, the research team will provide additional education about proper medication dosing, refer caregivers to the prescribed dose on the medication bottle and refer the caregiver back to their child's health care provider for further questions.

Secondary Outcomes: Secondary efficacy outcomes will include discharge medication adherence and caregiver medication comprehension assessed during the myCap follow-up survey. Adherence will be calculated based on caregiver report of first and last dose of medication (compared to documentation in electronic medical record) and if there were any missed doses or if the medication was never obtained from the pharmacy. Caregiver medication comprehension will be assessed using survey questions as was done in a previous study.[29] We will also assess the kind of medication teaching parents received in the hospital and which aspects of the medication teaching caregivers found the most useful. Caregiver follow-up survey is included in the application. We will also collect process and exploratory outcomes as detailed in Table 3.

Table 3. Outcomes and measures obtained during myCap follow-up survey		
**Primary Outcome**		
**Outcome**	**Measurement definition**	**How measurement will be obtained**
Medication Dose Accuracy	**Comprehension:** >20% deviation from caregiver *reported* dose as compared to dose in electronic health record (EHR)<br>**Adherence:** >20% deviation from *observed* dose as compared to dose in (EHR)	Reported dose accuracy measured via caregiver self-report during post-discharge video conference call<br><br>For observed dose, caregivers will be asked to measure out the prescribed volume using a syringe and submit a picture of the syringe via myCap
**Secondary Outcomes**		
Medication Name	**Comprehension:** Error in reporting medication name<br>**Adherence:** N/A	Caregiver self-report during myCap follow-up survey
Medication Indication	**Comprehension:** Error in reporting indication for medication<br>**Adherence:** N/A	Caregiver self-report during myCap follow-up survey
Medication Frequency	**Comprehension:** Any error in reporting frequency for medication<br>**Adherence:** Any error in reporting frequency for medication	Caregiver self-report during myCap follow-up survey
Medication Duration	**Comprehension:** Any error in reporting duration for medication<br>**Adherence:** Parent report of first and last dose of medication compared to prescribed duration	Caregiver self-report during myCap follow-up survey
Medication Side Effects	**Comprehension:** Unable to report any known side effects of the medication (credit for correctly identifying > 1 known side effort or reporting no known side effects)<br>**Adherence:** N/A	Caregiver self-report during myCap follow-up survey
**Exploratory Outcomes**		
Clinical Outcomes	Prevalence and type of major dosing errors<br><br>Unplanned visits to health care providers due to medication errors	Caregiver self-report during post-discharge video conference call<br><br>Review of EHR 30 days following discharge to assess for any unplanned health care visits

3.5 Data Analysis and Sample Size

Data analysis: Baseline characteristics of the intervention and control groups will be compared using *t* tests and χ^2 tests or Fisher exact tests, as appropriate. Wilcoxon rank-sum tests will be used if the data does not appear to be normally distributed. These tests will be performed to also compare those who did and did not complete the follow-up assessment.

Randomization will be done using block permutation at the time of enrollment. Study staff enrolling caregivers will not be blinded to group allocation. Caregivers will not explicitly be told what group they have been assigned. Study staff will be blinded to group allocation when assessing the dose in the syringe in the picture submitted during follow-up in order to determine the primary outcome of observed dosing accuracy. Two independent study staff will assess the picture to determine the dose. Any disagreement will be adjudicated by the study PI.

Table 4. Demographics of potentially eligible children, July 2018-June 2019 (N =1,742)	
**Characteristic**	**N (%)**
Age (y)	
0 to < 6 months	940 (54)
6 mos to <1yrs	183 (11)
12 mos to < 2 yrs	288(16)
2yrs to < 3yrs	205 (12)
3yrs to < 4 yrs	125 (7)
Sex	
Male	958 (55)
Female	784 (45)
Race/Ethnicity	
Non-Hispanic white	1098 (63)
Non-Hispanic black	244 (14)
Hispanic	296 (17)
Other	104 (6)

The primary outcome will be the percent difference in the observed dose measured as the difference in millimeters (mL) between the dose of medication in the caregiver submitted picture during follow-up assessment compared to the prescribed dose in the electronic health record divided by the prescribed dose. For example, if the observed dose in the caregiver submitted picture was 4.5 mL and the prescribed dose in the electronic health record was 3 mL then this would be a 50%

overdosing error. The observed dosing percent difference will be compared between the intervention and control groups as medians with interquartile ranges.

If we detect covariate imbalance in the two groups, we will use propensity score weighting and a multivariable linear regression model to conduct an adjusted analysis. Proposed covariates to include in the model are: caregiver health literacy, caregiver age, caregiver gender, caregiver race/ethnicity, and caregiver primary language. These covariates are likely to have the biggest impact on the primary outcome. The other covariates includes caregiver country of origin, socioeconomic status (i.e., binary outcome of do you have difficulty paying bills), employment status, education status, and martial status. Patient level covariates include child age, gender race/ethnicity, medical complexity, number of previous medications and previous hospitalizations in the last year and insurance type.

We will display our findings for the continuous outcome of observed medication dosing error using either box plots or violin plots for the intervention and control groups.

Given that prior literature in this area has examined medication dose error as a dichotomous variable using +/- 20% from the prescribed dose (this was felt to be a clinically meaningful dosing error), we will also examine and display our primary outcome in this way to allow for comparison to other studies. We will report relative risk reduction, absolute risk reduction and number needed to treat along with 95% confidence intervals for the primary outcome of both observed dosing accuracy and reported dosing accuracy.

Sample Size: Sample size estimates are based on the outcome of medication dosing accuracy. Using a conservative dosing error prevalence of 30% in the control arm,[2,4–6,15] and an expected dosing error prevalence of 10% in the intervention arm, we will need to enroll 72 subjects in each arm of the study to reject the null hypothesis that the dosing error prevalence in the control and intervention arms are equal with 80% power (2-sided alpha of 0.05). Accounting for 25-30% loss to follow-up, we plan to enroll a total of 200 subjects. From July 2018-June 2019, the pediatric hospital medicine service at Vanderbilt discharged 1,742 children <4 years of age, at least 50% of whom were discharged home on 1 or more medications (approximately 75 children per month), ensuring ample opportunities to achieve our required sample size (see Table 4).

3.6 Potential Limitations and Alternative Approaches for Aim 1

A potential challenge is slower than anticipated enrollment. If this occurs, we will extend our enrollment period and/or consider expanding our enrollment catchment to other clinical teams or older children (e.g. school age children). Although our study is powered to detect differences similar to what has been observed in prior studies, these assumptions may be inaccurate. Nonetheless, the study would still be very useful in measuring potential effect size estimates for future study.

4.0 Risk to Participants

All study subjects will receive standard of care for discharge medication counseling. Study subjects in the intervention group will receive additional and enhanced discharge medication counseling. This poses no greater than minimal risk to study participants. The potential benefit of this study would come be the development and implementation of more efficacious discharge medication counseling which will, hopefully, lead to a reduction in medication dosing errors and a decrease in patient harm.

5.0 Privacy/Confidentiality Issues

Privacy and confidentiality is of the highest priority, and all efforts will be made to keep personal information and research records private throughout these studies. Records and documents pertaining to the conduct of this study will be kept in HIPPA-compliant, secure, password protected databases (i.e., myCap and REDCap) or in locked files accessible only by a limited number of study personnel. All patient data will be coded using a

unique study ID. No PHI will be shared outside of the key study personnel at each institution and no PHI will be shared between institutions. The information from the research study may be published using aggregate results; however, no individual subject will be identified.

6.0 Reporting of Adverse Events or Unanticipated Problems Involving Risk to Participants

The PI and research study staff (with PI oversight) are responsible for ensuring protocol compliance, data integrity, and participant safety. This protocol presents minimal risks to participants and adverse events or other problems are not anticipated. In the unlikely event that such events occur, unanticipated problems involving risks to subjects or others will be reported immediately to the PI and the IRB and in writing within 5 days to the IRB and any appropriate funding and regulatory agencies. Annual reports will be submitted to the responsible IRBs summarizing study progress, adverse events, complaints about the research or withdrawals, and any protocol violations.

7.0 References

1. Smith MD, Spiller HA, Casavant MJ, Chounthirath T, Brophy TJ, Xiang H. Out-of-Hospital Medication Errors Among Young Children in the United States, 2002-2012. *PEDIATRICS*. 2014;134(5):867-876. doi:10.1542/peds.2014-0309

2. Frush KS, Luo X, Hutchinson P, Higgins JN. Evaluation of a Method to Reduce Over-the-Counter Medication Dosing Error. *ARCH PEDIATR ADOLESC MED*. 2004;158:5.

3. McMahon SR, Rimsza ME, Bay RC. Parents Can Dose Liquid Medication Accurately. *PEDIATRICS*. 1997;100(3):330-333. doi:10.1542/peds.100.3.330

4. Li SF, Lacher B, Crain EF. Acetaminophen and ibuprofen dosing by parents: *Pediatric Emergency Care*. 2000;16(6):394-397. doi:10.1097/00006565-200012000-00003

5. Simon HK. Over-the-counter Medications: Do Parents Give What They Intend to Give? *Arch Pediatr Adolesc Med*. 1997;151(7):654. doi:10.1001/archpedi.1997.02170440016003

6. Hyam E, Brawer M, Herman J, Zvieli S. What's in a Teaspoon? Underdosing with Acetaminophen in Family Practice. *Fam Pract*. 1989;6(3):221-223. doi:10.1093/fampra/6.3.221

7. Winnick S. How Do You Improve Compliance? *PEDIATRICS*. 2005;115(6):e718-e724. doi:10.1542/peds.2004-1133

8. Drotar D, Bonner MS. Influences on Adherence to Pediatric Asthma Treatment: A Review of Correlates and Predictors: *Journal of Developmental & Behavioral Pediatrics*. 2009;30(6):574-582. doi:10.1097/DBP.0b013e3181c3c3bb

9. HCUPnet, healthcare cost and utilizatin project 2018. Agency for Healthcare Research and Quality. Accessed August 5, 2020. https://hcupnet.ahrq.gov/

10. Feudtner C. Prevalence of Polypharmacy Exposure Among Hospitalized Children in the United States. *Arch Pediatr Adolesc Med*. 2012;166(1):9. doi:10.1001/archpediatrics.2011.161

11. Ratzan S, Parker R. National Library of Medicine current bibliographies in medicine: health literacy. In: *National Library of Medicine Current Bibliographies in Medicine: Health Literacy.*

12. Yin HS, Parker RM, Sanders LM, et al. Pictograms, Units and Dosing Tools, and Parent Medication Errors: A Randomized Study. *Pediatrics*. 2017;140(1):e20163237. doi:10.1542/peds.2016-3237

13. Yin HS, Dreyer BP, Moreira HA, et al. Liquid Medication Dosing Errors in Children: Role of Provider Counseling Strategies. *Academic Pediatrics*. 2014;14(3):262-270. doi:10.1016/j.acap.2014.01.003

14. Yin HS, Mendelsohn AL, Fierman A, van Schaick L, Bazan IS, Dreyer BP. Use of a Pictographic Diagram to Decrease Parent Dosing Errors With Infant Acetaminophen: A Health Literacy Perspective. *Academic Pediatrics*. 2011;11(1):50-57. doi:10.1016/j.acap.2010.12.007

15. Yin HS, Dreyer BP, van Schaick L, Foltin GL, Dinglas C, Mendelsohn AL. Randomized Controlled Trial of a Pictogram-Based Intervention to Reduce Liquid Medication Dosing Errors and Improve Adherence Among Caregivers of Young Children. *Arch Pediatr Adolesc Med*. 2008;162(9):814. doi:10.1001/archpedi.162.9.814

16. Rosman SL, Dorfman D, Suglia SF, Humphrey C, Silverstein M. Predictors of Prescription Filling After Visits to the Pediatric Emergency Department: *Pediatric Emergency Care*. 2012;28(1):22-25. doi:10.1097/PEC.0b013e31823ed6e4

17. Samuels-Kalow ME, Stack AM, Porter SC. Parental Language and Dosing Errors After Discharge From the Pediatric Emergency Department: *Pediatric Emergency Care*. 2013;29(9):982-987. doi:10.1097/PEC.0b013e3182a269ec

18. Yin HS, Dreyer BP, Ugboaja DC, et al. Unit of Measurement Used and Parent Medication Dosing Errors. *PEDIATRICS*. 2014;134(2):e354-e361. doi:10.1542/peds.2014-0395

19. Glick AF, Brach C, Yin HS, Dreyer BP. Health Literacy in the Inpatient Setting. *Pediatric Clinics of North America*. 2019;66(4):805-826. doi:10.1016/j.pcl.2019.03.007

20. The Joint Comission. A crosswalk of the national standards for culturally and linguistically appropriate services (CLAS) in health and health care to the Joint Commission Hospital Accreditation Standards 2014. Accessed August 5, 2020. https://www. jointcommission.org/assets/1/6/Crosswalk-_CLAS_-20140718.pdf

21. Morrison AK, Glick A, Yin HS. Health Literacy: Implications for Child Health. *Pediatrics in Review*. 2019;40(6):263-277. doi:10.1542/pir.2018-0027

22. Mallory LA, Osorio SN, Prato BS, et al. Project IMPACT Pilot Report: Feasibility of Implementing a Hospital-to-Home Transition Bundle. *Pediatrics*. 2017;139(3):e20154626. doi:10.1542/peds.2015-4626

23. Katz MG, Kripalani S, Weiss BD. Use of pictorial aids in medication instructions: A review of the literature. *American Journal of Health-System Pharmacy*. 2006;63(23):2391-2397. doi:10.2146/ajhp060162

24. Delp C, Jones J. Communicating Information to Patients: The Use of Cartoon Illustrations to Improve Comprehension of Instructions. *Academic Emergency Medicine*. 1996;3(3):264-270. doi:10.1111/j.1553-2712.1996.tb03431.x

25. Turner T, Cull WL, Bayldon B, et al. Pediatricians and Health Literacy: Descriptive Results From a National Survey. *Pediatrics*. 2009;124(Supplement 3):S299-S305. doi:10.1542/peds.2009-1162F

26. Harris PA, Taylor R, Thielke R, Payne J, Gonzalez N, Conde JG. Research electronic data capture (REDCap)—A metadata-driven methodology and workflow process for providing translational research informatics support. *Journal of Biomedical Informatics*. 2009;42(2):377-381. doi:10.1016/j.jbi.2008.08.010

Last Revision: 4/21/2022

27. Feudtner C, Feinstein JA, Zhong W, Hall M, Dai D. Pediatric complex chronic conditions classification system version 2: updated for ICD-10 and complex medical technology dependence and transplantation. *BMC Pediatr*. 2014;14(1):199. doi:10.1186/1471-2431-14-199

28. Weiss BD. Quick Assessment of Literacy in Primary Care: The Newest Vital Sign. *The Annals of Family Medicine*. 2005;3(6):514-522. doi:10.1370/afm.405

29. Glick AF, Farkas JS, Mendelsohn AL, et al. Discharge Instruction Comprehension and Adherence Errors: Interrelationship Between Plan Complexity and Parent Health Literacy. *The Journal of Pediatrics*. 2019;214:193-200.e3. doi:10.1016/j.jpeds.2019.04.052
