# Supplementary material for: Health Literacy–Informed Communication to Reduce Discharge Medication Errors in Hospitalized Children: A Randomized Clinical Trial
Source: JAMA Netw Open. 2024 Jan 16;7(1):e2350969. doi: 10.1001/jamanetworkopen.2023.50969 (PMC10792470; doi:10.1001/jamanetworkopen.2023.50969)
Supplement: Supplement 2. — eFigure. Example of Written, Pictogram-Based Medication Instruction Sheet eAppendix. Medication Indications, Adverse Effects, and Storage Information eMethods. Inverse Probability of Treatment Weighting Analysis eTable 1. Inverse Propensity Score Weighting of Characteristics of Children and Caregivers by Randomization Group for Primary Outcome (n=151) eTable 2. Comparison of Participants Included in Analysis and Those Lost to Follow-up or Withdrawn [file jamanetwopen-e2350969-s002.pdf]

Supplementary Online Content

Carroll AR, Johnson JA, Stassun JC, Greevy RA, Mixon AS, Williams DJ. Health literacy–informed communication to reduce discharge medication errors in hospitalized children: a randomized clinical trial. *JAMA Netw Open*. 2024;6(1):e2350969. doi:10.1001/jamanetworkopen.2023.50969

eFigure. Example of Written, Pictogram-Based Medication Instruction Sheet

eAppendix. Medication Indications, Adverse Effects, and Storage Information

eMethods. Inverse Probability of Treatment Weighting Analysis

eTable 1. Inverse Probability Score Weighting of Characteristics of Children and Caregivers by Randomization Group for Primary Outcome (n = 151)

eTable 2. Comparison of Participants Included in Analysis and Those Lost to Follow-up or Withdrawn

This supplementary material has been provided by the authors to give readers additional information about their work.

© 2024 Carroll AR et al. *JAMA Network Open*.

eFigure. Example of Written, Pictogram-Based Medication Instruction Sheet

Medicine Instructions for [child's name]

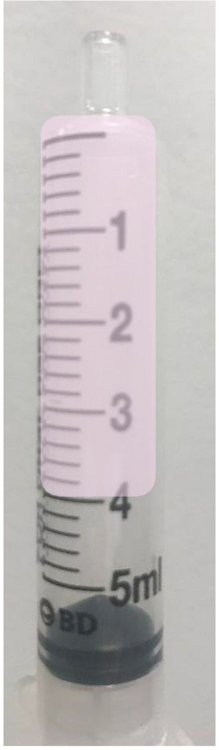

Medicine Name	Used for…	Instructions	Total Duration	Next dose due	Side Effects	Storage Instructions
**Cephalexin**<br>Commonly known as:<br>**Keflex**	Infection of the skin (Staphylococcal scalded skin syndrome)	Give **4 mL** by mouth **every 6 hours** (or 4 times a day)<br>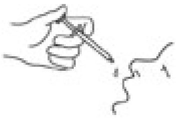	**6 days**	**1/19/2022**<br>at 3 pm	Diarrhea<br>Nausea<br>Vomiting<br>Rash	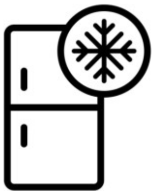<br>Keep in the fridge

© 2024 Carroll AR et al. *JAMA Network Open*.

eAppendix. Medication Indications, Adverse Effects, and Storage Information

Prednisolone:
Prednisolone is a steroid that helps treat inflammation.
Side Effects: increased appetite, difficulty sleeping, nausea if not taken with food
Storage: room temperature

Amoxicillin:
Amoxicillin is an antibiotic often used to treat a bacterial infection
Side Effects: diarrhea, nausea, vomiting, rash
Storage: In refrigerator (to improve taste) but can also be stored at room temperature

Augmentin (Amoxicillin and Clavulanate)
Augmentin (also called Amoxicillin and Clavulanate) is an antibiotic often used to treat a bacterial infection
Side Effects: diarrhea, nausea, vomiting, rash
Storage: In refrigerator

Keflex (Cephalexin):
Keflex (also called Cephalexin) is an antibiotic to often used to treat a bacterial infection
Side Effects: diarrhea, nausea, vomiting, rash
Storage: In refrigerator

Cleocin (Clindamycin):
Clindamycin is an antibiotic often used to treat a bacterial infection
Side Effects: stomachache, diarrhea, nausea, vomiting, rash
Storage: room temperature

Bactrim (Trimethoprim and Sulfamethoxazole):
Bactrim (Trimethoprim and Sulfamethoxazole) an antibiotic often used to treat a bacterial infection
Side Effects: stomachache, gassiness, diarrhea, nausea, vomiting, rash. *If a rash develops, call your doctor immediately.*
Storage: room temperature

Tylenol (Acetaminophen):
Acetaminophen (also called Tylenol) is a medication commonly used for pain or fever control.
Side effects: stomachache, vomiting. Do not take more than prescribed, liver damage is a toxic effect of acetaminophen overdose.
Storage: room temperature

Advil or Motrin (Ibuprofen):
Ibuprofen (also called Advil) is a non-steroid anti-inflammatory drug (NSAID) that is commonly used for pain or fever control.
Side effects: stomachache, vomiting, indigestion, heartburn. Do not take more than prescribed, kidney damage is a toxic effect of ibuprofen overdose.
Storage: room temperature

Allegra (Fexofenadine):
Allegra (also called Fexofenadine) is a medication used to prevent common symptoms of seasonal allergies.
Side effects: feeling tired, headache, vomiting
Storage: room temperature

Azithromycin
Azithromycin is an antibiotic to treat a bacterial infection
Side effects: stomachache, diarrhea, vomiting, rash
Storage: room temperature.

© 2024 Carroll AR et al. *JAMA Network Open*.

Benadryl (Diphenhydramine):
Benadryl (also called Diphenhydramine) is a medication used to help allergy symptoms such as itchiness and rash.
Side effects: feeling tired, feeling dizzy, dry mouth, stomachache
Storage: room temperature

Zyrtec (Cetirizine):
Zyrtec (also called Cetirizine) is a medication used to prevent common symptoms of seasonal allergies.
Side effects: feeling tired, feeling dizzy, stomachache
Storage: room temperature

Claritin (Loratadine):
Claritin (also called Loratadine) is a medication used to prevent common symptoms of seasonal allergies.
Side effects: feeling tired, headache
Storage: room temperature

Tamiflu (Oseltamivir):
Tamiflu (also known as Oseltamivir) is an antiviral medication which shortens the duration of flu symptoms.
Side effects: stomachache, vomiting
Storage: In refrigerator

Pepcid (Famotidine):
Pepcid (also called famotidine) is a medication treat gastroesophageal reflux disease (GERD)
Side effects: Abdominal pain, constipation, diarrhea, gas, nausea, vomiting
Storage: room temperature

Prevacid (Lansoprazole):
Prevacid (also called lansoprazole) is a medication treat gastroesophageal reflux disease (GERD)
Side effects: Constipation, diarrhea, upset stomach
Storage: In refrigerator

Neurontin (Gabapentin):
Neurontin (also called gabapentin) is a medication used to certain kinds of pain
Side effects: Increased sleepiness, muscle pain or weakness, tremors, diarrhea, dry mouth
Storage: In refrigerator

Katerzia (Amlodipine benzoate):
Amlodipine is a medication used to certain hypertension or high blood pressure
Side effects: peripheral edema
Storage: In refrigerator

Diflucan (fluconazole):
Fluconaolze is a medication used to treat certain fungal infections
Side effects: Nausea, abdominal pain, diarrhea
Storage: Room temperature OR refrigerator. Might taste better in the fridge

Keppra (Levetiracetam):
Keppra (also called Levetiracetam) is a medication used to treat seizures
Side effects: drowsiness, rash, vomiting
Storage: room temperature

Ciprofloxacin (Cipro):
Cipro is a medication used to treat infections
Side effects: inflammation of tendons, abdominal pain, diarrhea, nausea, vomiting
Storage: room temperature for 14 days
Special instructions: Do not give with milk or other dairy products or vitamins containing iron, zinc, or calcium

© 2024 Carroll AR et al. *JAMA Network Open*.

Omnicef (Cefdinir)
Cefdinir is a medication used to treat infections
Side effects: nausea, vomiting, diarrhea, rash
Storage: room temperature

Sezaby (Phenobarbital)
Phenobarbital is a medication to treat or prevent seizures
Side effects: increased sleepiness, rash
Storage: room temperature

Suprax (Cefixime)
Cefixime is a medication used to treat infections
Side effects: nausea, abdominal pain, diarrhea, rash
Storage: room temperature
Special instructions: Administer with foods to decreased GI distress

Carnitor (Levocarnitine)
Levocarnitine is a medication used to treat carnitine deficiency or other inborn errors of metabolism
Side effects: nausea, vomiting, diarrhea, allergic reaction like rash
Storage: room temperature
Special instructions: space doses evenly throughout the day and take during or following meals every 3 to 4 hours

Cefadroxil
Cefadroxil is a medication used to treat infection
Side effects: diarrhea
Storage: room temperature

Inderal or Hemangeol (Propranolol)
Propranolol is a medication used to treat blood rhythm problems or hemangiomas
Side effects: slow heart rate, low blood pressure, diarrhea, bronchospasm leading to wheezing or trouble breathing, low blood sugar
Storage: room temperature
Special instructions: Administer during or after a feeding to reduce risk for low blood sugar (hypoglycemia)

Levaquin (Levofloxacin)
Levofloxacin is a medication used to treat infections
Side effects: joint stiffness, dizziness, sleepiness, rash, diarrhea, irritated tendons
Storage: room temperature

Medication information obtained from Lexicomp online (Lexicomp, Inc., copyright 1978-2023) and reviewed by John A. Wright, PharmD, BCPPS inpatient pediatric pharmacist and the Monroe Carell Jr. Children's Hospital at Vanderbilt and clinical pharmacist for the Vanderbilt Pediatric Complex Care Program.

© 2024 Carroll AR et al. *JAMA Network Open*.

eMethods. Inverse Probability of Treatment Weighting Analysis

eMethods

Examination of Table 1 revealed some child and caregiver characteristics were imperfectly balanced, which is not unusual in a randomized trial of this size. Therefore, we performed a planned, post- hoc secondary analysis that adjusted for these chance imbalances in covariates via inverse probability of treatment weighting (IPTW). A propensity score (PS) for being in the treatment arm (i.e., intervention group) consisted of a logistic model of treatment by child age, sex, race, ethnicity, medication use, number of hospitalizations, presence of complex chronic conditions, insurance payer and medication class and caregiver age, sex, relationship to child, race, ethnicity, language, birthplace outside the U.S., education, difficulty paying bills, and Newest Vital Sign score. Weights were calculated as the reciprocal of the probability of being in the arm the subject was actually in conditioning on covariates (i.e., 1/PS for treated subjects and 1/(1-PS) for control subjects). Weighted Chi-squared and *t*-tests were performed in Stata® version 16.1, and statistical significance of the weighted *t*-tests were validated against weighted randomization tests.

© 2024 Carroll AR et al. *JAMA Network Open*.

eTable 1. Inverse Probability Score Weighting of Characteristics of Children and Caregivers by Randomization Group for Primary Outcome (n = 151)

Characteristic[a]	Intervention (n = 79)	Standard Counseling (n = 72)[b]	*P* value
**Children**			
Age, mean (SD), y	2.5	2.2	.81
Male sex	57	58	.90
Race			.56
Asian	9	9	
Black/African American	10	11	
Native Hawaiian/Pacific Islander	4	0	
White	71	71	
More than one race	12	12	
Prefer not to answer	6	2	
Hispanic/Latino ethnicity	23	23	.72
Uses medication(s) regularly[c]	37	33	.89
2+ hospitalizations in last year	19	16	.85
Complex Chronic Condition present	24	23	.98
Payer			
Government	62	62	.68
Private	37	37	
Medication Type			.97
Antimicrobial	90	87	
Anticonvulsants	2	2	
Steroid	1	3	
Gastrointestinal agents/antacids	2	1	
Other medications[d]	6	7	
**Caregivers**			
Age, mean (SD), y	31.5 (7.7)	31 (4.8)	.66
Female sex	95	95	>.99
Relationship to child, mother			.42
Mother	93	93	
Father	5	7	
Legal guardian	3	0	
Race			.40
Asian	1	0	
Black/African American	13	12	
Native Hawaiian/Pacific Islander	4	0	
White	79	79	
More than one race	1	3	
Prefer not to answer	2	7	
Hispanic/Latino ethnicity	19	18	.95
Spanish speaking	16	16	.90
Born outside the United States	22	22	.99
Education			.99
Less than high school diploma	18	15	
High school degree or equivalent	24	26	
Some College	31	29	
College	20	21	
Graduate degree	9	7	
Difficulty paying bills at home	22	24	.87
Newest Vital Sign (total score=6)			.96

© 2024 Carroll AR et al. *JAMA Network Open*.

Low (score 0-1)	15	16	
Marginal (score 2-3)	34	36	
Adequate (score 4-6)	51	48	

[a] Data given as % of participants unless otherwise indicated
[b] Race for one child is missing in the standard counseling group
[c] Uses medication(s) regularly refers to 1 or more daily scheduled medications at home
[d] Other medications includes cardiovascular agents and vitamins

© 2024 Carroll AR et al. *JAMA Network Open*.

eTable 2. Comparison of Participants Included in Analysis and Those Lost to Follow-up or Withdrawn (n = 197)

Characteristic[a]	Included in Primary Analysis (n = 151)	Lost to Follow-Up or Withdrawn (n = 46)	*P* value
**Children**			
Age, mean (SD), y	2.5 (2.1)	2.2 (1.9)	.36
Male sex	84 (56)	26(57)	>.99
Race[b]			.64
Asian	1 (1)	0	
Black/African American	17 (11)	9 (20)	
Native Hawaiian/Pacific Islander	3 (2)	0	
White	108 (72)	33 (72)	
More than one race	17 (11)	4 (9)	
Prefer not to answer	4 (3)	0	
Hispanic/Latino ethnicity	33 (22)	10 (22)	>.99
Uses medication(s) daily[c]	42 (28)	8 (17)	.074
2+ hospitalizations in last year	25 (17)	7 (15)	.22
Complex Chronic Condition present	34 (23)	12 (26)	.69
Payer			.55
Government	92 (61)	33 (72)	
Private	57 (38)	13 (28)	
No insurance	1 (1)	0	
Other	1 (1)	0	
Medication Type			.082
Antimicrobial	131 (88)	37 (80)	
Anticonvulsants	4 (3)	0	
Steroid	4 (3)	2 (4)	
Gastrointestinal agents/antacids	3 (2)	5 (11)	
Other medication[d]	9 (6)	2 (4)	
**Caregivers**			
Age, mean (SD), y	31.4 (6.5)	31.4 (6.2)	>.99
Female sex	142 (94)	43 (93)	>.99
Relationship to child, mother			
Mother	139 (92)	42 (91)	.47
Father	11 (7)	3 (7)	
Grandmother	0	1 (2)	
Legal guardian	1 (1)	0	
Race			.61
Asian	1 (1)	0	
Black/African American	19 (13)	9 (20)	
Native Hawaiian/Pacific Islander	3 (2)	0	
White	120 (79)	37 (80)	
More than one race	3 (2)	0	
Prefer not to answer	5 (3)	0	
Hispanic/Latino ethnicity	27 (18)	8 (17)	>.99
Spanish speaking	20 (13)	6 (13)	.81
Born outside the United States	30 (20)	9 (20)	>.99
Education			.39
Less than high school diploma	20 (13)	9 (20)	
High school degree or equivalent	39 (26)	15 (33)	
Some College	49 (32)	9 (20)	
College	30 (20)	8 (17)	

© 2024 Carroll AR et al. *JAMA Network Open*.

Graduate degree	13 (9)	5 (11)	
Difficulty paying bills at home	31 (21)	4 (9)	.079
Newest Vital Sign (total score=6)			.13
Low (score 0-1)	22 (15)	4 (9)	
Marginal (score 2-3)	49 (32)	23 (50)	
Adequate (score 4-6)	80 (53)	19 (41)	

[a] Data given as No. (%) of participants unless otherwise indicated
[b] Missing race for one child in included in primary analysis group
[c] Uses medication(s) regularly refers to 1 or more daily scheduled medications at home
[d] Other medication includes cardiovascular agents and vitamins

© 2024 Carroll AR et al. *JAMA Network Open*.
