# Supplementary material for: Health Literacy–Informed Communication to Reduce Discharge Medication Errors in Hospitalized Children: A Randomized Clinical Trial
Source: JAMA Netw Open. 2024 Jan 16;7(1):e2350969. doi: 10.1001/jamanetworkopen.2023.50969 (PMC10792470; doi:10.1001/jamanetworkopen.2023.50969)
Supplement: Supplement 3. — Data Sharing Statement [file jamanetwopen-e2350969-s003.pdf]

Data Sharing Statement

Carroll. Health Literacy–Informed Communication to Reduce Discharge Medication Deviations in Hospitalized Children. *JAMA Netw Open*. Published January 16, 2024. doi:10.1001/jamanetworkopen.2023.50969

Data
Data available: No
